# Impact of a Single Oral Acute Dose of Aflatoxin B_1_ on Liver Function/Cytokines and the Lymphoproliferative Response in C57Bl/6 Mice

**DOI:** 10.3390/toxins9110374

**Published:** 2017-11-17

**Authors:** Angélica Tieme Ishikawa, Elisa Yoko Hirooka, Paula Leonello Alvares e Silva, Ana Paula Frederico Rodrigues Loureiro Bracarense, Karina Keller Marques da Costa Flaiban, Claudia Yuri Akagi, Osamu Kawamura, Marcio Carvalho da Costa, Eiko Nakagawa Itano

**Affiliations:** 1 Department of Pathological Sciences, State University of Londrina, P.O. Box 10.011, Londrina 86057-970, Paraná, Brazil; paulaleonello@gmail.com (P.L.A.S.); yuri.akagi@gmail.com (C.Y.A.); 2 Department of Food Science and Technology, State University of Londrina, P.O. Box 10.011, Londrina 86057-970, Paraná, Brazil; hirooka@uel.br; 3 Department of Preventive Veterinary Medicine, State University of Londrina, P.O. Box 10.011, Londrina 86051-970, Paraná, Brazil; anapaula@uel.br (A.P.F.R.L.B.); kkflaiban@uel.br (K.K.M.d.C.F.); 4 Food Hygiene Laboratory, Faculty of Agriculture, Kagawa University, Miki-cho 761-0795, Kagawa, Japan; kawamura@ag.kagawa-u.ac.jp; 5 Department of Veterinary Biomedicine, University of Montreal, 3200 Rue Sicotte, St-Hyacinthe, QC J2S 2M2, Canada; marcio.costa@umontreal.ca

**Keywords:** cytokines, inflammatory response, immunosuppression, mycotoxin

## Abstract

Aflatoxin B_1_ (AFB_1_), a mycotoxin found in food and feed, exerts harmful effects on humans and animals. The liver is the earliest target of AFB_1_, and its effects have been evaluated in animal models exposed to acute or chronic doses. Considering the possibility of sporadic ingestion of AFB_1_-contaminated food, this study investigated the impact of a single oral dose of AFB_1_ on liver function/cytokines and the lymphoproliferative response in mice. C57BL/6 mice were treated with a single oral AFB_1_ dose (44, 442 or 663 μg AFB_1_/kg of body weight) on the first day. Liver function (ALT, γ-GT, and total protein), cytokines (IL-4, IFN-γ, and IL-17), histopathology, and the spleen lymphoproliferative response to mitogens were evaluated on the 5th day. Although AFB_1_ did not produce any significant changes in the biochemical parameters, 663 μg AFB_1_/kg-induced hepatic upregulation of IL-4 and IFN-γ, along with liver tissue injury and suppression of the lymphoproliferative response to ConA (*p* < 0.05). In conclusion, a single oral dose of AFB_1_ exposure can induce liver tissue lesions, liver cytokine modulation, and immune suppression in C57BL/6 mice.

## 1. Introduction

Aflatoxins (AFs) are mycotoxins produced by *Aspergillus flavus* and *A. parasiticus* that contaminate agricultural commodities under harvest and post-harvest conditions. Human and animal health issues caused by ingestion of food contaminated with AFs are considered a permanent risk and a worldwide problem [1,2]. The mammalian liver, an early target of AFs, is the major drug-detoxifying organ, responsible for the metabolic activation and elimination of toxic components [3].

Among the AFs, aflatoxin B_1_ (AFB_1_) is generally predominant and is considered the most toxic analog [4]. Ingestion of AFB_1_-contaminated products can result in immunosuppressive, teratogenic, mutagenic, and carcinogenic effects. The International Agency for Research on Cancer classifies AFB_1_ as Group 1, i.e., carcinogenic to humans [5,6]. Furthermore, AFB_1_ exerts many immunotoxic effects, ranging from alterations in innate immunity or antigen-presenting cells [7,8,9] to changes in adaptive immunity, resulting in a reduced number of circulating lymphocytes, the inhibition of lymphocyte blastogenesis, and the alteration of cytokine expression in animals of various species [9,10].

In studies of the different effects and impact of AFB_1_ exposure, animals such as mice have been exposed to acute or chronic AFB_1_ doses for an extended period [11,12,13,14]. Considering the possibility of sporadic ingestion of AFB_1_-contaminated food, this study investigated the impact of a single oral dose of AFB_1_ (with low doses; <to ~1% of the median lethal dose (LD_50_)) on liver function/cytokines and the lymphoproliferative response in C57Bl/6 mice.

## 2. Results

### 2.1. Evaluation of the Effect of Aflatoxin B_1_ on Serum ALT, γ-GT, and Total Protein Levels

Oral administration of AFB_1_ did not produce any significant change in the ALT and γ-GT serum enzymes or protein levels (*p* > 0.05). Additionally, the vehicle group treated with saline:ethanol (95:5) presented similar results to those obtained for the water-treated controls (*p* > 0.05) (Table 1).

Means values within column with different superscript letters were statistically significant (*p* < 0.05), as determined through Tukey’s test. The reference values for mice serum enzymes are 23.00 ± 4.92 units/L of ALT, 7.57 ± 4.2 units/L of γ-GT, and of 5.07 ± 0.2 g/dL of total protein [15,16].

### 2.2. Evaluation of Aflatoxin B_1_ on Histological Lesions in Liver Tissue

**Figure 1:**
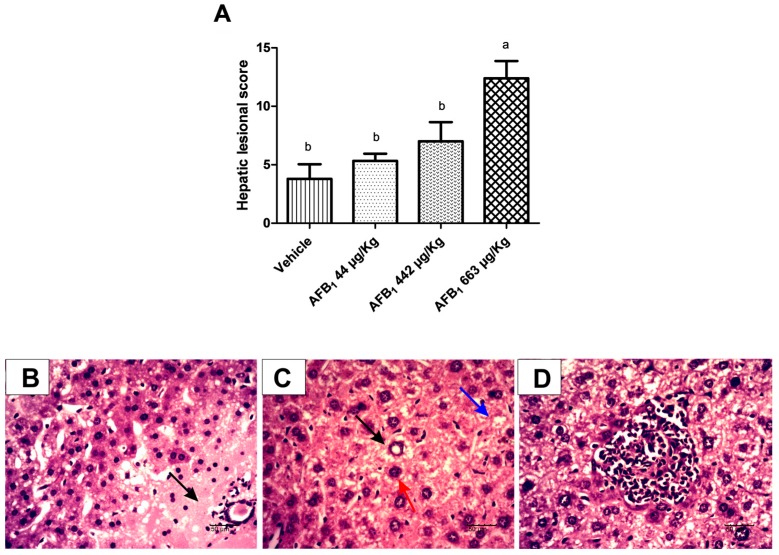
Effect of aflatoxin B_1_ on the livers of mice exposed to 44 μg, 442 μg, and 663 μg of AFB_1_/kg b.w. at 5 days after exposure. (**A**) Lesional score. The data are expressed as the mean ± SD, *n* = 5. Means without a common letter were statistically significant (*p* < 0.05), as demonstrated by Tukey’s test. The liver lesions observed in animals treated with AFB_1_ were (**B**) focal necrosis of hepatocytes (arrow), HE, 40×, 50 μm; (**C**) vacuolar hepatocyte degeneration (nuclear (black arrow) and cytoplasmic (blue arrow)) and megalocytosis (red arrow), HE, 40×, 50 μm; and (**D**) centrolobular inflammatory infiltrate, HE, 40×, 50 μm.

Cytoplasmic hepatocyte vacuolation, megalocytosis, nuclear vacuolation, inflammatory infiltrate, and necrosis were present in the liver of treated animals; the last two lesions were more frequently observed. Lesions recorded in the liver were considered moderate to severe in mice treated with AFB_1_. A significant increase in the lesional score was observed in animals exposed to 663 μg of AFB_1_/kg of body weight (b.w.) compared with animals exposed to vehicle (*p* = 0.001 (Figure 1). Animals treated with 663 μg of AFB_1_/kg of b.w. showed more pronounced intensity of megalocytosis, nuclear vacuolation, and necrosis than animals treated with vehicle, whereas the main lesions in the animals treated with 44 and 442 μg of AFB_1_/kg of b.w. were necrosis.

### 2.3. Effects of Aflatoxin B_1_ on Cytokine Expression in the Liver

**Figure 2:**
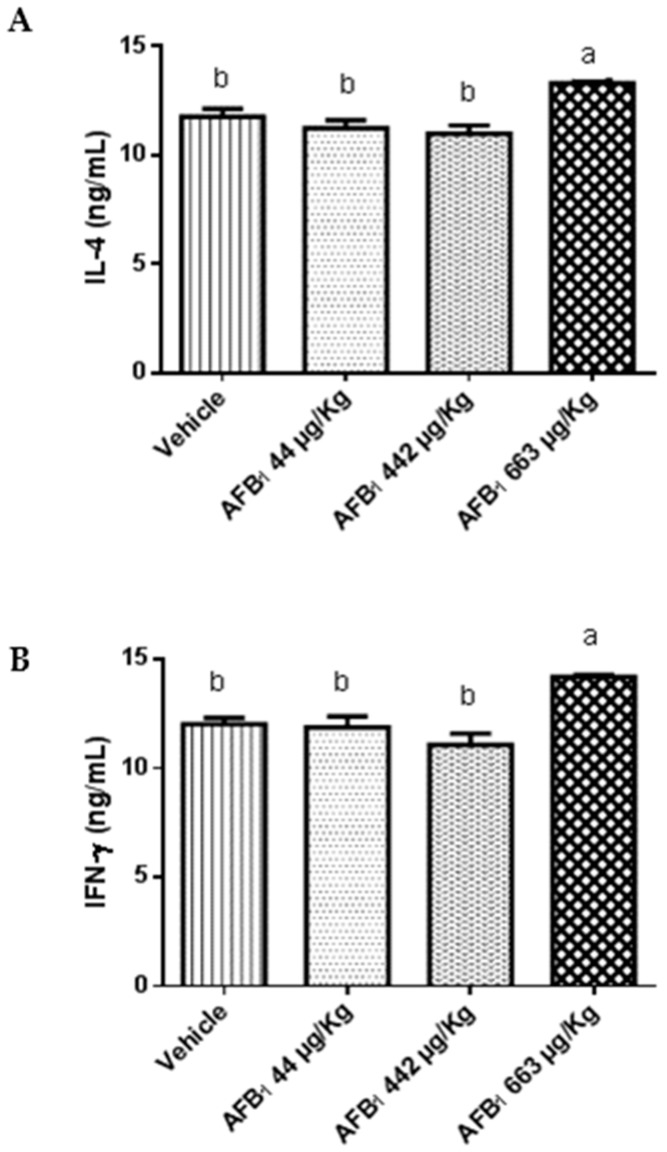

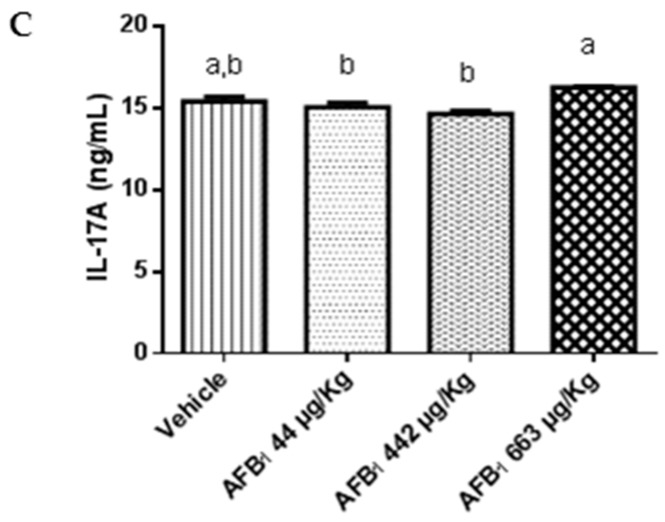
Effect of aflatoxin B_1_ on the expression levels of (**A**) interleukin 4 (IL-4), (**B**) interferon γ (IFN-γ), and (**C**) interleukin 17 (IL-17) in the liver at 5 days post-aflatoxin exposure. Data are expressed as the mean ± SD, *n* = 5. ^a,b^ columns with different superscript letters were statistically significant (*p* < 0.05), as determined by Tukey’s test.

Mice treated with 663 μg of AFB_1_/kg b.w. showed significant upregulation of IL-4 and IFN-γ (*p* = 0.002) compared with the vehicle group (Figure 2). There was no difference in the IL-17 cytokine levels between animals treated with AFB_1_ and untreated animals (*p* > 0.05). In contrast, there were differences between animals treated with 44 and 442 μg of AFB_1_/kg of b.w. and animals treated with 663 μg of AFB_1_/kg of b.w. (*p* < 0.05).

### 2.4. Effects of Aflatoxin B_1_ on Lymphoproliferation Assay

**Figure 3:**
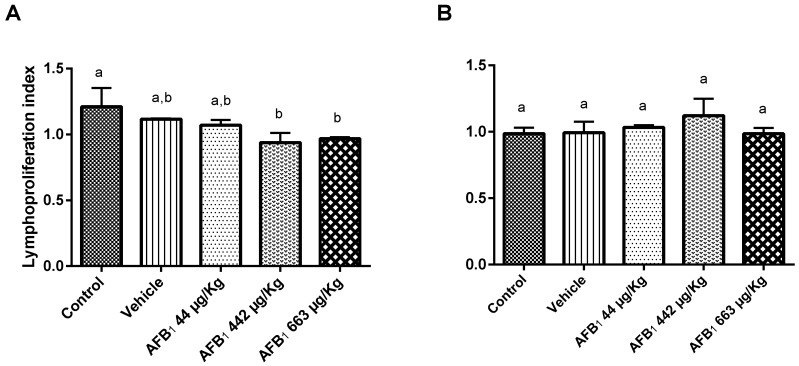
Effects of aflatoxin B_1_ on mouse splenocyte proliferative responses in the presence of (**A**) concanavalin A or (**B**) lipopolysaccharide 5 days after aflatoxin treatment. Significance levels were determined based on the comparison of experimental animal data with control animal data. The data are expressed as the mean ± standard deviation (SD) of the proliferative index (optical density (OD) of the test well/OD of control well) for five animals. ^a,b^ columns with different superscript letters were statistically significant (*p* < 0.05), as determined by Tukey’s test.

Significant suppression of the proliferative response was observed for concanavalin A (ConA)-stimulated lymphocytes under treatment with 442 and 663 μg AFB_1_/kg of b.w. (*p* < 0.05). The control animals showed a lymphoproliferation index of 1.21 ± 0.14, whereas the experimental animals treated with doses of 442 μg and 663 μg AFB_1_/kg of b.w. showed indexes of 0.94 ± 0.07 and 0.97 ± 0.01, respectively. However, no significant depression of the proliferative assay was observed in lipopolysaccharide (LPS)-stimulated lymphocytes when the data were compared with those of the control animals (Figure 3).

## 3. Discussion

Among mycotoxins, AFs are of major concern worldwide in terms of their risks to human and animal health. Their harmful effects have generally been demonstrated by administering repeated AF doses over long periods of exposure in animal models. In this study, we investigated the effects of exposure to single oral doses of AFB_1_ in C57Bl/6 mice. AFB_1_ did not have a major impact on the investigated hepatic biochemical parameters in the mice 5 days after administration. It is possible that the time of sampling was too long and the measurement of liver functions levels in earlier days was be different in animals treated with AFB_1_ compared to the control group. However, such results are in accordance with the results of a study conducted by Almeida et al. [15], who did not detect differences in the alkaline phosphatase levels in the serum of C57Bl/6 mice after 168 h of AF treatment (60 mg/kg animal weight). Moreover, our results are consistent with those observed by Baptista et al. [17], who fed albino rats 400 μg AFB_1_/kg of b.w. over 28 days and did not observe any significant differences in ALT, AST, ALP, or γ-GT enzyme activities or albumin levels.

The sensitivity degree and toxicity of AFB_1_ varies between species due to differences in its biotransformation. Some animals, such as sheep, dogs, pigs, and rats, are considered extremely susceptible to AFB_1,_ whereas others, such as monkeys, chickens and mice, are considered resistant species [18]. The LD_50_ described in the literature for mice is variable, with values ranging from 9 to 60 mg of AFB_1_/kg of b.w. [15]. The doses of AFB_1_ used in this experiment (44, 442, and 663 μg/kg of b.w.) might appear high when applied to humans; however, aflatoxicosis cases have been reported to occur at similar or higher consumption levels of AFB_1_ [19,20].

Changes in the hepatic cellular architecture and organization were detected by histopathological analysis. The extent of liver damage was directly correlated with the concentration of AFB_1_ and the duration of the exposure [17]. In this study, a 7.7-fold increase in the lesional score was observed in AFB_1_-exposed animals. Notably, this study constitutes the first evaluation of the effects of a subclinical, single AFB_1_ exposure in mice, and the hepatic lesions observed here are consistent with those observed in other studies that used different doses or frequencies of exposure [21].

In the present study, upregulation of the production of the hepatic inflammatory cytokine IFN-γ, along with increased expression of the anti-inflammatory cytokine IL-4, were observed with higher doses of AFB_1_. There is no consensus regarding the cytokine responses induced by AF exposure [22,23,24]. Here, the difference obtained in anti- and pro-inflammatory cytokines levels might be due to time, a single and higher dose (663 μg of AFB_1_/kg of b.w.), or different organs and species. Because the evaluations performed in this study were at early stages (5 days) prior to the adaptive response, increases in IL-4 and IFN-γ expression were attributed to innate immune cell activation. Additionally, many innate immune cell populations produce IL-17 in response to stress, injury, or pathogens [25]. Our results indicate that IL-17 levels were significantly different among the highest and lowest doses of AFB_1_, but no differences were detected between the treated and control groups. Thus, even a single dose of AFB_1_ might induce hepatic cytokine immunomodulation, but whether all of the cytokines evaluated are involved in hepatic injury remains uncertain, and further studies are required.

AFB_1_ has a selective effect on cell-mediated immunity, with a relatively weak effect on the humoral immune system [21]. Consistently, in this study, we detected the inhibitory effects of AFB_1_ on ConA-stimulated lymphoblastogenesis (ConA is a lectin widely used as a polyclonal T-cell activator) but not on LPS-stimulated lymphoblastogenesis. Reddy & Sharma [26] reported the inhibitory effects of AFB_1_ on both LPS- and ConA-stimulated lymphoblastogenesis in animals exposed to low, repeated doses of AFB_1_. The difference in the LPS response detected in this investigation could be due to the use of a single dose instead of repeated doses.

According to a review by Peraica et al. [27], the acute hepatotoxic effects of AFs recorded in humans have mostly been observed among adults in rural populations with poor nutritional levels. The same authors cited the case of a young woman who ingested a total of 5.5 mg of AFB_1_ over 2 days, and whose laboratory examinations were normal and suggested that the hepatotoxicity of AFB_1_ might be lower in well-nourished persons.

In this study, the biochemical parameters of hepatic functions were not altered in mice exposed to AFB_1_. However, several other important parameters, such as liver tissue injury, cytokine levels, and cellular responses, were altered even with a single dose. Moreover, in a previous study, we verified that a single AFB_1_ exposure induces changes in the gut microbiota in C57Bl/6 mice [28].

It is important to investigate the effects of a single dose of AFs due to the possibility of sporadic ingestion of aflatoxin-contaminated food. In this study, harmful effects were observed even with one low oral dose of AFB_1_ (~1% of the median lethal dose (LD_50_) for mice [26]). These effects might be temporary but could contribute to exacerbation in cases of patients with liver disease or other diseases associated with immunosuppression, requiring further studies.

## 4. Conclusions

In conclusion, even a single, oral, low dose of AFB_1_ can induce liver tissue lesions, liver cytokine modulation and immune suppression in C57BL/6 mice.

## 5. Material and Methods

### 5.1. Aflatoxin B_1_ Standard, Dose Criteria, and Duration of Exposure

The AFB_1_ standard from *Aspergillus flavus* (A663, Sigma, St. Louis, MO, USA) was quantified according to the methods indicated by Instituto Adolfo Lutz [29]. The molar absorptivity of AFB_1_ considered for calculating AFB_1_ concentrations in methanol was 21,800 at 360 nm. The AFB_1_ solution was dried under gaseous N_2_, and the standard was dissolved in a 95:5 saline:ethanol solution for experiments.

The lowest AFB_1_ dose (44 μg/kg of b.w.) was calculated based on the maximum level allowed (5 μg/kg) in both rice and beans [30,31], according to the average bean (183 g/day) and rice (160 g/day) consumption levels, which are components of the traditional diet in Brazil [32]. The highest tested AFB_1_ dose (663 μg/kg of b.w.) was based on the chronic AFB_1_ dose used in other studies [13,33] and represents approximately 1% of the LD_50_ for mice. The duration of exposure was based on the plasma half-life of AFB_1_ in male rats, which is approximately 92 h [34].

### 5.2. Animals, Housing, and Experimental Design

A total of 25 male C57Bl/6 mice (10 weeks of age, average weight: 22.55 ± 0.89 g) were obtained from the University of São Paulo—Ribeirão Preto City, Brazil. C57Bl/6 mice were selected for this study because they are highly susceptible to the acute effects of aflatoxin B_1_ [15]. The mice were acclimatized for 3 weeks and housed in polyethylene boxes with a bedding of wood shavings. They were maintained under standard conditions, which included a temperature of approximately 25 °C with a regular 12 h light/12 h dark cycle. All mice were given standard rodent pellet food and water *ad libitum*.

The experimental design used in this study was randomized with five repetitions (each animal represented one repetition) for each group. Group 1 consisted of untreated control mice; Group 2 received only the vehicle (saline:ethanol, 95:5) on the first day; Group 3 received a single dose of 44 μg AFB_1_/kg b.w. on the first day; Group 4 received a single dose of 442 μg AFB_1_/kg b.w.; and Group 5 received a single dose of 663 μg AFB_1_/kg b.w. AFB_1_ suspended in saline:ethanol (95:5) was administered via oral gavage (0.1 mL per 10 g of body weight). After 5 days, the animals were bled and euthanized, and their organs (liver and spleen) were then removed.

Biochemical parameters were analyzed from the serum, and a lymphoproliferation assay was performed using splenocytes. This study was approved by Committee of Animal Ethics of State University of Londrina (CEUA n° 26362.2014.65 process, 18 December 2014).

### 5.3. Measurements of Liver Function from Serum

ALT and γ-GT enzymes and total protein levels in serum were evaluated with a Dimension^®^ Clinical Chemistry System (Siemens, Newark, NJ, USA).

### 5.4. Histopathological Analysis

Liver samples were fixed in 10% buffered formalin solution, dehydrated in increasing alcohol concentrations, and embedded in paraffin for histological analysis. The tissue samples were sectioned at 5-μm thickness, stained with hematoxylin and eosin (HE), and mounted with coverslips. Histological changes were evaluated using an adapted tissue score based on the intensity and severity of lesions as previously described by Gerez et al. [35]. Briefly, the criteria used to establish the lesional score were hepatocyte megalocytosis, inflammatory infiltrate, hepatocyte nuclear vacuolation, hepatocyte cytoplasmic vacuolation, and necrosis. The extent of each lesion was scored as follows, megalocytosis (the mean of five fields per histological section): 1–10 = 0, 1–20 = 1+, 2–30 = 2+, and 3–40 = 3+; inflammatory infiltrate and nuclear vacuolation (histological section): absent = 0, 1 = 1+, and >2 = 2+; hepatic cell vacuolation (histological section): mild = 0, and moderate = 1+; and necrosis (histological section): absent = 0, mild = 1+, moderate = 2+, and severe = 3+. For each type of lesion, the score of the extent was multiplied by the severity factor [35].

### 5.5. Cytokine Assays

The liver samples were macerated in an adjusted volume of 0.1 M phosphate buffered saline (PBS), pH 7.4 (1:5000 dilution). The IL-4, IFN-γ, and IL-17 levels in the liver were determined by commercial sandwich ELISA kits (BioSource International, Inc., Camarillo, CA, USA), according to the manufacturer’s instructions.

### 5.6. Lymphoproliferation Assay

The spleens were removed from the mice aseptically, and their erythrocytes were lysed with Tris-ammonium chloride solution. In 96-well flat-bottom culture plates, 100 μL of the splenocytes (1 × 10^5^ cells/mL) from each mouse were cultured in duplicate wells in RPMI 1640 medium (containing l-glutamine and, 10% fetal calf serum), with 0.5 μg/mL LPS (Sigma, St. Louis, MO, USA) or with 0.5 μg/mL ConA (Gibco Life Technologies, Grand Island, NY, USA). The cells were cultured for 84 h at 37 °C with 5% CO_2_, and 100 μL of RPMI medium and 10 μL of 3-(4,5-dimethylthiazol-2-ly)-2,5-diphenyltetrazolium bromide (5 mg/mL MTT in PBS, Sigma, St. Louis, MO, USA) were then added to each well. The plates were then further incubated for 4 h at 37 °C, and formazan crystals were subsequently solubilized by adding 200 μL of dimethyl sulfoxide (DMSO). The optical density was subsequently measured with an ELISA microplate reader (iMarkTM, Bio-Rad, Hercules, CA, USA) at 550 nm, and the proliferation index (P. I.) of the stimulated/nonstimulated cells was calculated in duplicate [36].

### 5.7. Statistical Analysis

The biochemical parameter and lymphoproliferative assay data were analyzed using Statistica software (version 7.0, 2004, Stat Soft, Tulsa, OK, USA) and are presented as the mean ± standard deviation. Before the analysis, homogeneity of variance (Levene’s test) and the normality of the data distribution (Shapiro-Wilk’s test) were tested. One-way analysis of variance (ANOVA) followed by Tukey’s test was performed, and *p* values < 0.05 were considered statistically significant.

## Figures and Tables

**Table 1:** Effects of aflatoxin B_1_ on alanine aminotransferase (ALT), gamma glutamyl transpeptidase (γ-GT), and total protein levels in mice serum five days after a single oral dose of aflatoxin B_1_.

Group	Parameters		
	ALT (U/L)	γ-GT (U/L)	Total Protein (g/dL)
Control	26.67 ± 9.37 ^a^	6.83 ± 0.75 ^a^	5.63 ± 0.49 ^a^
Vehicle	43.25 ± 15.52 ^a^	6.5 ± 0.71 ^a^	5.46 ± 0.54 ^a^
AFB_1_ 44 μg/kg	37.33 ± 8.64 ^a^	6.3 ± 1.03 ^a^	5.83 ± 0.27 ^a^
AFB_1_ 442 μg/kg	28.33 ± 7.23 ^a^	5.50 ± 0.71 ^a^	5.12 ± 0.34 ^a^
AFB_1_ 663 μg/kg	22.80 ± 3.70 ^a^	7.00 ± 2.00 ^a^	5.46 ± 0.30 ^a^

^a^ *p* < 0.05.

## Abbreviations

AFB_1_	aflatoxin B_1_
AFs	aflatoxins
ALP	alkaline phosphatase
ALT	alanine aminotransferase
AST	aspartate aminotransferase
b.w.	body weight
ConA	concanavalin A
DMSO	dimethyl sulfoxide
ELISA	enzyme-linked immunosorbent assay
LD_50_	median lethal dose
LPS	lipopolysaccharide
MTT	3-(4,5-dimethylthiazol-2-yl)-2,5-diphenyltetrazolium bromide
P. I.	proliferation index
PBS	phosphate buffered saline
RPMI	Roswell Park Memorial Institute
γ-GT	γ-glutamyl transpeptidase
